# Reply – Clinical Spectrum of Drug-Induced Movement Disorders: A Study of 97 Patients

**DOI:** 10.5334/tohm.591

**Published:** 2020-12-28

**Authors:** Anjali Chouksey, Sanjay Pandey

**Affiliations:** 1 Department of Neurology, Govind Ballabh Pant Postgraduate Institute of Medical Education and Research, New Delhi, IN

**Keywords:** Drug-induced movement disorder, Dopamine receptor blocker agents, Tardive syndromes

We thank Gupta and colleagues for their comments on our recently published article on drug-induced movement disorders (DIMDs) [1,2]. We will like to clarify certain relevant points raised by them. Firstly, the most common mechanism of drug-induced tremors is the enhancement of physiological tremor, which is low-amplitude, high-frequency tremor at rest and during the action that is often asymptomatic [3]. A temporal relation to the start of pharmacotherapy and dose-response relation is useful in making the diagnosis of drug-induced or drug- exacerbated tremor. Nevertheless, we agree with Gupta et al that sometimes it can be challenging to differentiate drug-induced tremor from essential tremor, however, lack of tremor progression after stoppage of the suspected drug can give a clue to drug-induced etiology. Secondly, the most common antiepileptic drugs (AEDs) to cause movement disorder in our study were valproate, phenytoin, and levetiracetam. We agree with the comments of Gupta et al that the levetiracetam induced dyskinesia has rarely been described in the literature [4]. However, 22 % of patients of AED associated DIMD in our study were on polytherapy, including other AEDs or dopamine receptor blocking agents (DRBA). In such patients, the contribution of each drug in the pathogenesis of DIMD is difficult to access. Thirdly, Gupta et al pointed out the entity called tardive tremor, which occurs after long-term use of neuroleptic medication; however, this category is not included in the DSM-5 criteria of DIMD. We also had five patients in our study who developed tremor after the intake of neuroleptics. Finally, we agree with the comments of Gupta et al that there is a need to raise awareness among the emergency room physicians and general practitioners about movement disorders as a possible side effect of different pharmacological agents including DRBAs and non-DRBAs. Thus, our intent behind this study was to call the attention of physicians towards DIMD patients in whom the cessation of medication can help to ameliorate the involuntary movement and avoid unnecessary workup.
